# Supplementary material for: Crafting for a better MAGIC: systematic design and test for Multiparental Advanced Generation Inter-Cross population
Source: G3 (Bethesda). 2021 Aug 18;11(11):jkab295. doi: 10.1093/g3journal/jkab295 (PMC8527519; doi:10.1093/g3journal/jkab295)
Supplement: jkab295_Supplementary_Data [file jkab295_supplementary_data.docx]

**Supplementary Materials**

for

**Crafting for a better MAGIC: systematic design and test for multiparental advanced generation inter-cross population.**

Chin Jian Yang

Rodney N. Edmondson

Hans-Peter Piepho

Wayne Powell

Ian Mackay

# Supplementary Tables

## Table S1. Number of funnels in a partial balanced set or full design.

In P2 design, a balanced design is defined where all n founders are present equally in the funnels and all founder pairings are present equally at each level of crosses. The number of funnels required for a partial balanced set is $n-1$ and for full design is $n!/2^{n-1}$. In NP2 design, a balanced design is less strictly defined where only all founders are present equally in the funnels, without the latter requirement in P2. The number of funnels required for a partial balanced set is $x$ where $x$ must be the smallest positive integer that satisfies $\left( x\cdot n_{0} \right)/n mod 1=0$ and $n_{0}$ is the next highest P2 $n$. Number of funnels in a full design is provided up to $n=8$ as the number of funnels in a full design for higher $n$ is impractical.

| n | set | full |  | n | set | full |  | n | set | full |  | n | set | full |
| --- | --- | --- | --- | --- | --- | --- | --- | --- | --- | --- | --- | --- | --- | --- |
| 3 | 3 | 3 |  | 35 | 35 | NA |  | 67 | 67 | NA |  | 99 | 99 | NA |
| 4 | 3 | 3 |  | 36 | 9 | NA |  | 68 | 17 | NA |  | 100 | 25 | NA |
| 5 | 5 | 240 |  | 37 | 37 | NA |  | 69 | 69 | NA |  | 101 | 101 | NA |
| 6 | 3 | 855 |  | 38 | 19 | NA |  | 70 | 35 | NA |  | 102 | 51 | NA |
| 7 | 7 | 945 |  | 39 | 39 | NA |  | 71 | 71 | NA |  | 103 | 103 | NA |
| 8 | 7 | 315 |  | 40 | 5 | NA |  | 72 | 9 | NA |  | 104 | 13 | NA |
| 9 | 9 | NA |  | 41 | 41 | NA |  | 73 | 73 | NA |  | 105 | 105 | NA |
| 10 | 5 | NA |  | 42 | 21 | NA |  | 74 | 37 | NA |  | 106 | 53 | NA |
| 11 | 11 | NA |  | 43 | 43 | NA |  | 75 | 75 | NA |  | 107 | 107 | NA |
| 12 | 3 | NA |  | 44 | 11 | NA |  | 76 | 19 | NA |  | 108 | 27 | NA |
| 13 | 13 | NA |  | 45 | 45 | NA |  | 77 | 77 | NA |  | 109 | 109 | NA |
| 14 | 7 | NA |  | 46 | 23 | NA |  | 78 | 39 | NA |  | 110 | 55 | NA |
| 15 | 15 | NA |  | 47 | 47 | NA |  | 79 | 79 | NA |  | 111 | 111 | NA |
| 16 | 15 | NA |  | 48 | 3 | NA |  | 80 | 5 | NA |  | 112 | 7 | NA |
| 17 | 17 | NA |  | 49 | 49 | NA |  | 81 | 81 | NA |  | 113 | 113 | NA |
| 18 | 9 | NA |  | 50 | 25 | NA |  | 82 | 41 | NA |  | 114 | 57 | NA |
| 19 | 19 | NA |  | 51 | 51 | NA |  | 83 | 83 | NA |  | 115 | 115 | NA |
| 20 | 5 | NA |  | 52 | 13 | NA |  | 84 | 21 | NA |  | 116 | 29 | NA |
| 21 | 21 | NA |  | 53 | 53 | NA |  | 85 | 85 | NA |  | 117 | 117 | NA |
| 22 | 11 | NA |  | 54 | 27 | NA |  | 86 | 43 | NA |  | 118 | 59 | NA |
| 23 | 23 | NA |  | 55 | 55 | NA |  | 87 | 87 | NA |  | 119 | 119 | NA |
| 24 | 3 | NA |  | 56 | 7 | NA |  | 88 | 11 | NA |  | 120 | 15 | NA |
| 25 | 25 | NA |  | 57 | 57 | NA |  | 89 | 89 | NA |  | 121 | 121 | NA |
| 26 | 13 | NA |  | 58 | 29 | NA |  | 90 | 45 | NA |  | 122 | 61 | NA |
| 27 | 27 | NA |  | 59 | 59 | NA |  | 91 | 91 | NA |  | 123 | 123 | NA |
| 28 | 7 | NA |  | 60 | 15 | NA |  | 92 | 23 | NA |  | 124 | 31 | NA |
| 29 | 29 | NA |  | 61 | 61 | NA |  | 93 | 93 | NA |  | 125 | 125 | NA |
| 30 | 15 | NA |  | 62 | 31 | NA |  | 94 | 47 | NA |  | 126 | 63 | NA |
| 31 | 31 | NA |  | 63 | 63 | NA |  | 95 | 95 | NA |  | 127 | 127 | NA |
| 32 | 31 | NA |  | 64 | 63 | NA |  | 96 | 3 | NA |  | 128 | 127 | NA |
| 33 | 33 | NA |  | 65 | 65 | NA |  | 97 | 97 | NA |  |  |  |  |
| 34 | 17 | NA |  | 66 | 33 | NA |  | 98 | 49 | NA |  |  |  |  |

## Table S2. Published MAGIC populations.

Most, if not all, of the MAGIC populations that have been described in either published literature or pre-prints are listed as of March 17, 2021.

| Species | n | Design | Pop. | Data Avail. | References |
| --- | --- | --- | --- | --- | --- |
| *Arabidopsis thaliana* | 19 | Semi-structured | 703 | Yes | [Gnan *et al.* (2014)](https://doi.org/10.1534/genetics.114.170746) |
|  | 8 | Semi-structured | 532 | No | [Huang *et al.* (2011)](https://doi.org/10.1073/pnas.1100465108) |
| *Brassica napus* (Rapeseed) | 8 | Basic | 680 | No | [Zhao *et al.* (2017)](https://doi.org/10.7505/j.issn.1007-9084.2017.02.002) |
| *Brassica juncea* (Chinese mustard) | 8 | Basic | 408 | No | [Yan *et al.* (2020)](https://doi.org/10.1111/pbr.12820) |
| *Gossypium hirsutum* (Cotton) | 11 | Unstructured | 550 | No | [Islam *et al.* (2016)](https://doi.org/10.1186/s12864-016-3249-2) |
|  | 12 | Semi-structured | 258 | No | [Li *et al.* (2016)](https://doi.org/10.4238/gmr15048759) |
|  | 8 | Basic | 960 | No | [Huang *et al.* (2018)](https://doi.org/10.1007/s00438-018-1419-4) |
| *Fragaria x ananassa* (Strawberry) | 6 | Unstructured | 338 | No | [Wada *et al.* (2017)](https://doi.org/10.1270/jsbbs.17009) |
| *Glycine max* (Soybean) | 8 | Partial, 3 funnels | 764 | No | [Shivakumar *et al.* (2018)](http://doi.org/10.18520/cs/v114/i04/906-908) |
| *Hordeum vulgare* (Barley) | 8 | Basic | 533 | No | [Sannemann *et al.* (2015)](https://doi.org/10.1007/s11032-015-0284-7) |
|  | 32 | Basic | 324 | No | [Bülow *et al.* (2019)](https://doi.org/10.5073/JfK.2019.11.02) |
|  | 8 | Basic | 122 | No | [Novakazi *et al.* (2020)](https://doi.org/10.3390/genes11121512) |
|  | 8 | Basic | 29 | No | [Novakazi *et al.* (2020)](https://doi.org/10.3390/genes11121512) |
|  | 8 | Basic | 81 | No | [Novakazi *et al.* (2020)](https://doi.org/10.3390/genes11121512) |
|  | 8 | Basic | 303 | No | [Novakazi *et al.* (2020)](https://doi.org/10.3390/genes11121512) |
| *Oryza sativa* ssp. *indica* (Rice) | 8 | Basic | 245 | No | [Li et al. (2013)](https://doi.org/10.1007/s10681-013-0879-1) |
|  | 4 | Basic | 532 | No | [Meng *et al.* (2016)](https://doi.org/10.3835/plantgenome2015.10.0109) |
|  | 4 | Basic | 271 | No | [Meng *et al.* (2016)](https://doi.org/10.3835/plantgenome2015.10.0109) |
|  | 8 | Basic | 268 | No | [Meng *et al.* (2016)](https://doi.org/10.3835/plantgenome2015.10.0109) |
|  | 8 | Partial, 35 funnels | 1316 | Yes | [Raghavan *et al.* (2017)](https://doi.org/10.1534/g3.117.042101) |
|  | 8 | Partial, 35 funnels, X1 | 144 | No | [Descalsota *et al.* (2018)](https://doi.org/10.3389/fpls.2018.01347) |
| *Oryza sativa* ssp. *japonica* (Rice) | 8 | Partial, 35 funnels | 500 | No | [Bandillo *et al.* (2013)](https://doi.org/10.1186/1939-8433-6-11) |
| *Oryza sativa* ssp. *indica x japonica* (Rice) | 12 | Semi-structured | 206 | No | [Li et al. (2014)](http://doi.org/10.5958/j.2348-7542.15.1.004) |
|  | 8 | Partial, 2 funnels | 981 | No | [Ogawa *et al.* (2018)](https://doi.org/10.1038/s41598-018-22657-3) |
|  | 16 | Semi-structured | 1027 | No | [Zaw *et al.* (2019)](https://doi.org/10.1038/s41598-019-55357-7) |
|  | 4 | Basic | 247 | No | [Han *et al.* (2020)](https://doi.org/10.1007/s00122-019-03440-y) |

*Table continued on the next page.*

*Table continued from the previous page.*

| Species | n | Design | Pop. | Data Avail. | References |
| --- | --- | --- | --- | --- | --- |
| *Solanum lycopersicum* (Tomato) | 8 | Basic | 397 | Yes | [Pascual *et al.* (2015)](https://doi.org/10.1111/pbi.12282) |
|  | 8 | Basic | 400 | No | [Campanelli *et al.* (2019)](https://doi.org/10.3390/agronomy9030119) |
| *Sorghum bicolor* (Sorghum) | 29 | Unstructured | 200 | Yes | [Ongom and Ejeta (2018)](https://doi.org/10.1534/g3.117.300248) |
| *Triticum aestivum* (Bread wheat) | 4 | Basic | 1579 | No | [Huang *et al.* (2012)](https://doi.org/10.1111/j.1467-7652.2012.00702.x) |
|  | 8 | Partial, 210 funnels | 643 | Yes | [Mackay *et al.* (2014)](https://doi.org/10.1534/g3.114.012963) |
|  | 60 | Unstructured | 1000 | No | [Thépot *et al.* (2015)](https://doi.org/10.1534/genetics.114.169995) |
|  | 8 | Basic, X1 | 394 | Yes | [Stadlmeier *et al.* (2018)](https://doi.org/10.3389/fpls.2018.01825) |
|  | 8 | Basic | 910 | Yes | [Sannemann *et al.* (2018)](https://dx.doi.org/10.1186/s12864-018-4915-3) |
|  | 8 | Partial, 311 funnels | 2381 | Yes | [Shah *et al.* (2019)](https://doi.org/10.1101/594317) |
|  | 8 | Partial, 313 funnels, X2 | 286 | Yes | [Shah *et al.* (2019)](https://doi.org/10.1101/594317) |
|  | 8 | Partial, 313 funnels, X3 | 745 | Yes | [Shah *et al.* (2019)](https://doi.org/10.1101/594317) |
|  | 16 | Partial, 15 funnels | 504 | Yes | [Scott *et al.* (2021)](https://doi.org/10.1101/2020.09.15.296533) |
| *Triticum durum* (Durum wheat) | 4 | Basic | 338 | No | [Milner *et al.* (2016)](https://doi.org/10.1111/pbi.12424) |
| *Vicia faba* (Faba bean) | 11 | Unstructured | 188 | No | [Sallam and Martsch (2015)](https://doi.org/10.1007/s10709-015-9848-z) |
|  | 4 | Basic | 1200 | No | [Khazaei *et al.* (2018)](https://doi.org/10.1017/S1479262118000242) |
| *Vigna unguiculata* (Cowpea) | 8 | Basic | 305 | Yes | [Huynh *et al.* (2018)](https://doi.org/10.1111/tpj.13827) |
| *Zea mays* (Maize) | 9 | Semi-structured | 529 | Yes | [Dell’Acqua *et al.* (2015)](https://doi.org/10.1186/s13059-015-0716-z) |
|  | 4 | Basic | 120 | No | [Mahan *et al.* (2018)](https://doi.org/10.2135/cropsci2017.07.0450) |
|  | 4 | Basic, X1 | 234 | No | [Mahan *et al.* (2018)](https://doi.org/10.2135/cropsci2017.07.0450) |
|  | 4 | Basic, X2 | 106 | No | [Mahan *et al.* (2018)](https://doi.org/10.2135/cropsci2017.07.0450) |
|  | 4 | Basic, X3 | 545 | No | [Mahan *et al.* (2018)](https://doi.org/10.2135/cropsci2017.07.0450) |
|  | 8 | Basic, X6 | 672 | No | [Jiménez-Galindo *et al.* (2019)](https://doi.org/10.1186/s12870-019-2052-z) |

Note: The founder marker data in the bread wheat MAGIC population from Stadlmeier et al. (2018) is not publicly available. Since the rice founders in Li et al. (2014) are unspecified, this MAGIC population is placed under *indica* x *japonica* group.

## Table S3. Number of informative recombinations in wheat-UK8 (full dataset).

The number of informative recombinations (NR) is calculated for both simulated and actual wheat-UK8 dataset. Note: recombinant inbred line (RIL), Morgan (M).

| Chr | NR/RIL | | NR/RIL/M | |
| --- | --- | --- | --- | --- |
|  | sim | actual | sim | actual |
| 1A | 8.42 | 3.37 | 3.53 | 1.41 |
| 1B | 12.94 | 4.64 | 3.69 | 1.32 |
| 1D | 4.44 | 1.78 | 3.38 | 1.36 |
| 2A | 9.36 | 5.18 | 3.61 | 2.00 |
| 2B | 13.64 | 3.58 | 3.59 | 0.94 |
| 2D | 6.50 | 2.97 | 3.27 | 1.49 |
| 3A | 11.13 | 5.40 | 3.60 | 1.75 |
| 3B | 10.45 | 4.29 | 3.69 | 1.51 |
| 3D | 5.59 | 2.03 | 2.87 | 1.05 |
| 4A | 7.94 | 3.32 | 3.64 | 1.52 |
| 4B | 8.43 | 3.03 | 3.63 | 1.30 |
| 4D | 3.95 | 1.36 | 3.14 | 1.08 |
| 5A | 11.07 | 5.42 | 3.53 | 1.73 |
| 5B | 11.38 | 3.23 | 3.65 | 1.04 |
| 5D | 6.42 | 2.58 | 3.17 | 1.28 |
| 6A | 10.42 | 4.23 | 3.66 | 1.49 |
| 6B | 9.57 | 2.67 | 3.64 | 1.02 |
| 6D | 5.44 | 1.03 | 2.53 | 0.48 |
| 7A | 14.24 | 5.19 | 3.69 | 1.35 |
| 7B | 10.19 | 3.78 | 3.55 | 1.32 |
| 7D | 6.35 | 2.28 | 2.91 | 1.05 |
| All | 187.88 | 71.39 | 3.48 | 1.32 |

## Table S4. Number of informative recombinations in wheat-DE8 (full dataset).

The number of informative recombinations (NR) is calculated for both simulated and actual wheat-DE8a dataset. Note: recombinant inbred line (RIL), Morgan (M).

| Chr | NR/RIL | | NR/RIL/M | |
| --- | --- | --- | --- | --- |
|  | sim | actual | sim | actual |
| 1A | 5.03 | 3.26 | 3.60 | 2.34 |
| 1B | 5.96 | 5.11 | 3.54 | 3.04 |
| 1D | 4.80 | 2.45 | 2.73 | 1.39 |
| 2A | 6.07 | 5.46 | 3.36 | 3.03 |
| 2B | 5.96 | 4.84 | 3.53 | 2.87 |
| 2D | 4.22 | 4.14 | 3.12 | 3.07 |
| 3A | 6.00 | 5.72 | 3.46 | 3.30 |
| 3B | 4.97 | 5.35 | 3.58 | 3.85 |
| 3D | 4.07 | 2.54 | 2.61 | 1.63 |
| 4A | 5.36 | 3.2 | 3.44 | 2.06 |
| 4B | 3.97 | 2.35 | 3.46 | 2.05 |
| 4D | 3.53 | 0.81 | 2.36 | 0.54 |
| 5A | 5.06 | 6.06 | 3.57 | 4.28 |
| 5B | 7.21 | 4.27 | 3.33 | 1.97 |
| 5D | 4.71 | 1.88 | 2.57 | 1.03 |
| 6A | 4.93 | 4.4 | 3.58 | 3.20 |
| 6B | 4.44 | 3.6 | 3.63 | 2.94 |
| 6D | 4.03 | 2.73 | 2.67 | 1.81 |
| 7A | 7.31 | 5.85 | 3.51 | 2.81 |
| 7B | 6.08 | 3.95 | 3.43 | 2.23 |
| 7D | 5.57 | 3.31 | 2.64 | 1.56 |
| All | 109.29 | 81.27 | 3.21 | 2.39 |

## Table S5. Proportions of individual recombinant haplotypes in five MAGIC population designs.

The mean proportions (standard deviations in parentheses) are shown for each recombinant haplotype and design. Two-ways recombinant haplotypes in design 5 are highlighted.

| Rec. hap. | Design 1 | Design 2 | Design 3 | Design 4 | Design 5 |
| --- | --- | --- | --- | --- | --- |
| 1_2 | 0.0030 (0.0019) | 0.0031 (0.0020) | 0.0027 (0.0018) | 0.0036 (0.0036) | 0.0069 (0.0206) |
| 1_3 | 0.0032 (0.0021) | 0.0028 (0.0020) | 0.0031 (0.0022) | 0.0031 (0.0030) | 0.0048 (0.0108) |
| 1_4 | 0.0030 (0.0020) | 0.0029 (0.0025) | 0.0025 (0.0021) | 0.0033 (0.0028) | 0.0058 (0.0104) |
| 1_5 | 0.0032 (0.0019) | 0.0030 (0.0021) | 0.0038 (0.0038) | 0.0036 (0.0034) | 0.0033 (0.0036) |
| 1_6 | 0.0035 (0.0021) | 0.0029 (0.0024) | 0.0031 (0.0022) | 0.0027 (0.0027) | 0.0029 (0.0034) |
| 1_7 | 0.0031 (0.0018) | 0.0033 (0.0027) | 0.0023 (0.0016) | 0.0030 (0.0027) | 0.0033 (0.0033) |
| 1_8 | 0.0029 (0.0019) | 0.0034 (0.0028) | 0.0030 (0.0024) | 0.0032 (0.0033) | 0.0033 (0.0036) |
| 2_1 | 0.0029 (0.0018) | 0.0031 (0.0023) | 0.0028 (0.0016) | 0.0030 (0.0036) | 0.0092 (0.0212) |
| 2_3 | 0.0027 (0.0018) | 0.0027 (0.0021) | 0.0032 (0.0026) | 0.0036 (0.0035) | 0.0030 (0.0083) |
| 2_4 | 0.0030 (0.0019) | 0.0029 (0.0025) | 0.0026 (0.0018) | 0.0030 (0.0028) | 0.0022 (0.0057) |
| 2_5 | 0.0035 (0.0020) | 0.0027 (0.0020) | 0.0027 (0.0017) | 0.0037 (0.0032) | 0.0021 (0.0029) |
| 2_6 | 0.0031 (0.0020) | 0.0030 (0.0025) | 0.0024 (0.0019) | 0.0032 (0.0030) | 0.0023 (0.0032) |
| 2_7 | 0.0030 (0.0019) | 0.0029 (0.0025) | 0.0040 (0.0036) | 0.0031 (0.0031) | 0.0018 (0.0023) |
| 2_8 | 0.0029 (0.0017) | 0.0036 (0.0028) | 0.0034 (0.0026) | 0.0038 (0.0035) | 0.0022 (0.0024) |
| 3_1 | 0.0027 (0.0018) | 0.0027 (0.0022) | 0.0030 (0.0021) | 0.0028 (0.0026) | 0.0022 (0.0056) |
| 3_2 | 0.0028 (0.0018) | 0.0029 (0.0021) | 0.0032 (0.0023) | 0.0034 (0.0034) | 0.0009 (0.0015) |
| 3_4 | 0.0028 (0.0019) | 0.0030 (0.0024) | 0.0031 (0.0024) | 0.0037 (0.0040) | 0.0054 (0.0170) |
| 3_5 | 0.0031 (0.0020) | 0.0032 (0.0024) | 0.0033 (0.0024) | 0.0033 (0.0028) | 0.0023 (0.0028) |
| 3_6 | 0.0032 (0.0019) | 0.0028 (0.0025) | 0.0033 (0.0030) | 0.0036 (0.0031) | 0.0025 (0.0037) |
| 3_7 | 0.0031 (0.0020) | 0.0031 (0.0024) | 0.0030 (0.0018) | 0.0036 (0.0035) | 0.0023 (0.0035) |
| 3_8 | 0.0032 (0.0021) | 0.0031 (0.0024) | 0.0029 (0.0026) | 0.0037 (0.0032) | 0.0026 (0.0032) |
| 4_1 | 0.0029 (0.0017) | 0.0027 (0.0022) | 0.0032 (0.0028) | 0.0032 (0.0032) | 0.0037 (0.0103) |
| 4_2 | 0.0029 (0.0021) | 0.0034 (0.0027) | 0.0024 (0.0014) | 0.0029 (0.0027) | 0.0049 (0.0109) |
| 4_3 | 0.0027 (0.0019) | 0.0032 (0.0027) | 0.0032 (0.0024) | 0.0032 (0.0030) | 0.0076 (0.0190) |
| 4_5 | 0.0030 (0.0016) | 0.0029 (0.0023) | 0.0028 (0.0024) | 0.0034 (0.0033) | 0.0028 (0.0030) |
| 4_6 | 0.0029 (0.0017) | 0.0027 (0.0021) | 0.0034 (0.0033) | 0.0033 (0.0034) | 0.0028 (0.0031) |
| 4_7 | 0.0028 (0.0017) | 0.0032 (0.0025) | 0.0029 (0.0024) | 0.0032 (0.0033) | 0.0023 (0.0028) |
| 4_8 | 0.0031 (0.0019) | 0.0028 (0.0026) | 0.0035 (0.0026) | 0.0034 (0.0036) | 0.0032 (0.0035) |
| 5_1 | 0.0030 (0.0018) | 0.0030 (0.0023) | 0.0034 (0.0030) | 0.0028 (0.0026) | 0.0033 (0.0036) |
| 5_2 | 0.0029 (0.0017) | 0.0028 (0.0023) | 0.0026 (0.0017) | 0.0036 (0.0029) | 0.0023 (0.0029) |
| 5_3 | 0.0030 (0.0018) | 0.0029 (0.0024) | 0.0029 (0.0025) | 0.0036 (0.0037) | 0.0027 (0.0030) |
| 5_4 | 0.0029 (0.0015) | 0.0026 (0.0020) | 0.0029 (0.0025) | 0.0030 (0.0033) | 0.0028 (0.0032) |
| 5_6 | 0.0027 (0.0018) | 0.0030 (0.0023) | 0.0027 (0.0016) | 0.0033 (0.0031) | 0.0074 (0.0211) |
| 5_7 | 0.0028 (0.0018) | 0.0034 (0.0029) | 0.0027 (0.0016) | 0.0034 (0.0030) | 0.0045 (0.0112) |
| 5_8 | 0.0028 (0.0017) | 0.0026 (0.0021) | 0.0032 (0.0028) | 0.0029 (0.0024) | 0.0057 (0.0110) |
| 6_1 | 0.0032 (0.0019) | 0.0031 (0.0022) | 0.0030 (0.0023) | 0.0035 (0.0030) | 0.0029 (0.0031) |
| 6_2 | 0.0031 (0.0020) | 0.0031 (0.0025) | 0.0024 (0.0018) | 0.0028 (0.0029) | 0.0021 (0.0028) |
| 6_3 | 0.0026 (0.0018) | 0.0028 (0.0024) | 0.0038 (0.0029) | 0.0031 (0.0028) | 0.0026 (0.0032) |
| 6_4 | 0.0030 (0.0019) | 0.0029 (0.0024) | 0.0034 (0.0032) | 0.0030 (0.0024) | 0.0029 (0.0031) |
| 6_5 | 0.0033 (0.0016) | 0.0032 (0.0022) | 0.0026 (0.0019) | 0.0032 (0.0029) | 0.0088 (0.0238) |
| 6_7 | 0.0029 (0.0018) | 0.0031 (0.0025) | 0.0028 (0.0023) | 0.0037 (0.0033) | 0.0025 (0.0063) |
| 6_8 | 0.0031 (0.0020) | 0.0028 (0.0027) | 0.0028 (0.0022) | 0.0030 (0.0028) | 0.0043 (0.0102) |
| 7_1 | 0.0026 (0.0017) | 0.0034 (0.0026) | 0.0024 (0.0016) | 0.0030 (0.0028) | 0.0029 (0.0028) |
| 7_2 | 0.0029 (0.0018) | 0.0027 (0.0025) | 0.0048 (0.0043) | 0.0035 (0.0031) | 0.0018 (0.0024) |
| 7_3 | 0.0034 (0.0023) | 0.0029 (0.0024) | 0.0030 (0.0019) | 0.0034 (0.0028) | 0.0023 (0.0028) |
| 7_4 | 0.0029 (0.0017) | 0.0031 (0.0024) | 0.0028 (0.0021) | 0.0030 (0.0031) | 0.0023 (0.0030) |
| 7_5 | 0.0031 (0.0018) | 0.0033 (0.0024) | 0.0029 (0.0017) | 0.0030 (0.0029) | 0.0039 (0.0094) |
| 7_6 | 0.0030 (0.0020) | 0.0029 (0.0026) | 0.0027 (0.0022) | 0.0037 (0.0035) | 0.0030 (0.0068) |
| 7_8 | 0.0028 (0.0020) | 0.0025 (0.0021) | 0.0025 (0.0018) | 0.0033 (0.0029) | 0.0059 (0.0182) |
| 8_1 | 0.0029 (0.0019) | 0.0030 (0.0026) | 0.0034 (0.0026) | 0.0037 (0.0035) | 0.0032 (0.0037) |
| 8_2 | 0.0027 (0.0018) | 0.0027 (0.0021) | 0.0031 (0.0025) | 0.0036 (0.0029) | 0.0023 (0.0030) |
| 8_3 | 0.0030 (0.0018) | 0.0035 (0.0027) | 0.0032 (0.0024) | 0.0034 (0.0033) | 0.0025 (0.0027) |
| 8_4 | 0.0028 (0.0019) | 0.0027 (0.0023) | 0.0031 (0.0025) | 0.0041 (0.0042) | 0.0031 (0.0035) |
| 8_5 | 0.0031 (0.0022) | 0.0032 (0.0027) | 0.0033 (0.0028) | 0.0036 (0.0035) | 0.0047 (0.0111) |
| 8_6 | 0.0030 (0.0021) | 0.0032 (0.0027) | 0.0028 (0.0024) | 0.0035 (0.0032) | 0.0043 (0.0117) |
| 8_7 | 0.0030 (0.0020) | 0.0030 (0.0024) | 0.0024 (0.0015) | 0.0033 (0.0038) | 0.0063 (0.0180) |

# Supplementary Figures


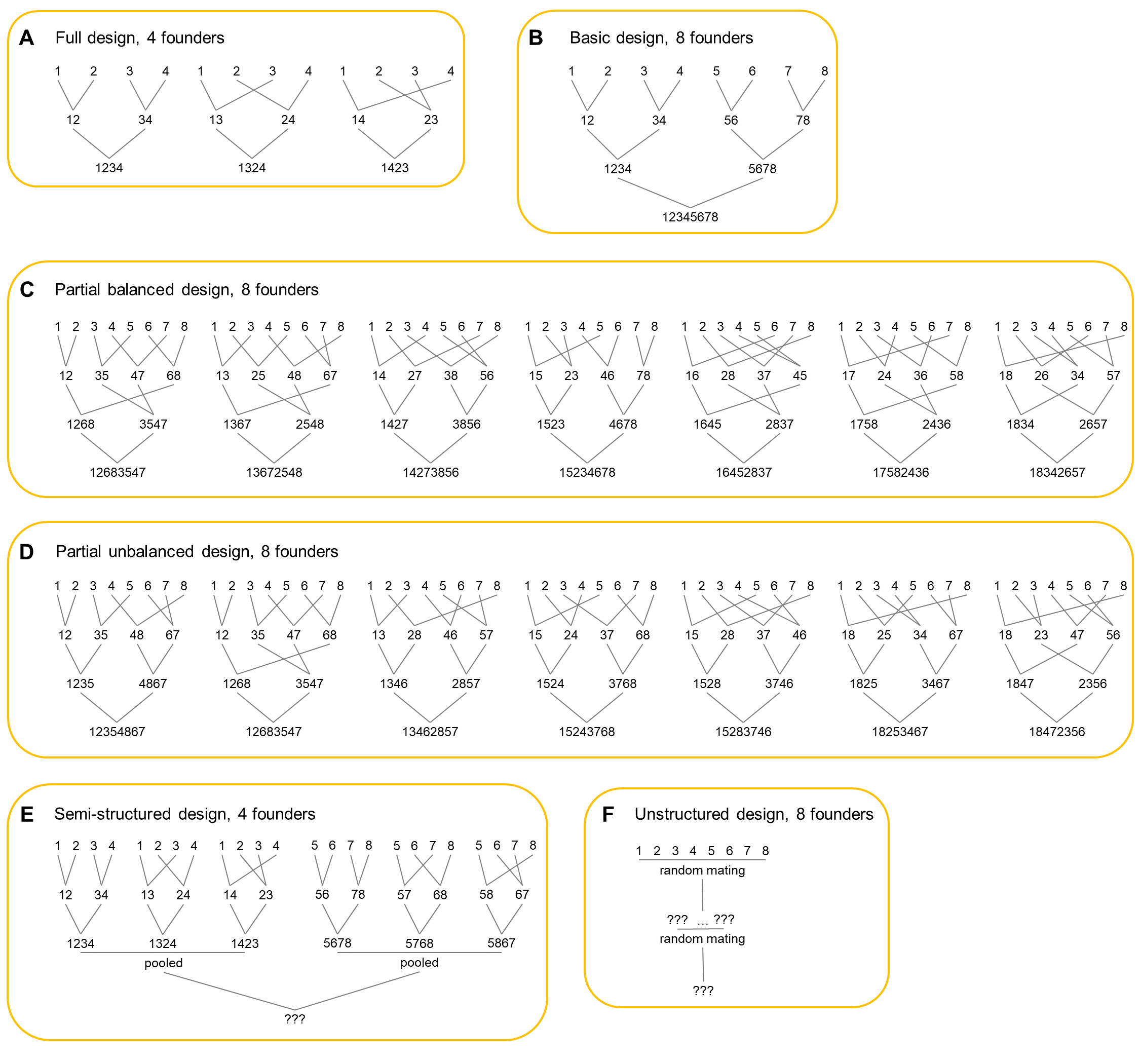


## Figure S1. Examples of MAGIC population designs.

[**A**] Full design with 4 founders. [**B**] Basic design with 8 founders. [**C**] Partial balanced design with 7 funnels and 8 founders. [**D**] Partial unbalanced design with 7 funnels and 8 founders. [**E**] Semi-structured design with 8 founders. [**F**] Unstructured design with 8 founders.


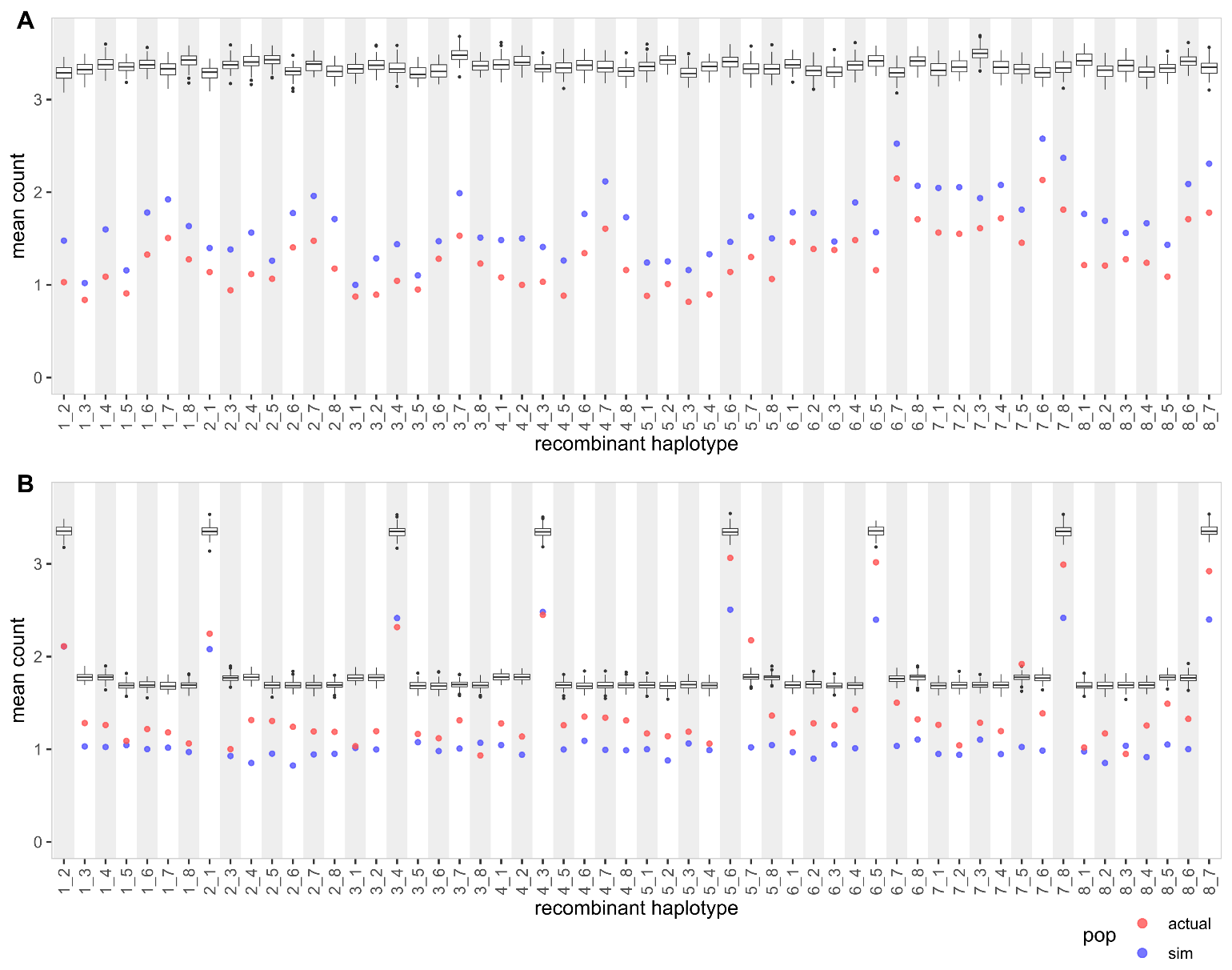


## Figure S2. Distributions of recombinant haplotypes in two wheat MAGIC populations (full datasets).

[**A**] Plot shows mean count of each recombinant haplotype in a single RIL in wheat-UK8 (full dataset with 643 RILs and 18,599 markers). The boxplot shows mean count from true founder genotypes (100 simulated iterations). The red and blue points show mean count from inferred founder genotypes. [**B**] Plot shows mean count of each recombinant haplotype in a single RIL in wheat-DE8 (full dataset with 910 RILs and 7,579 markers).


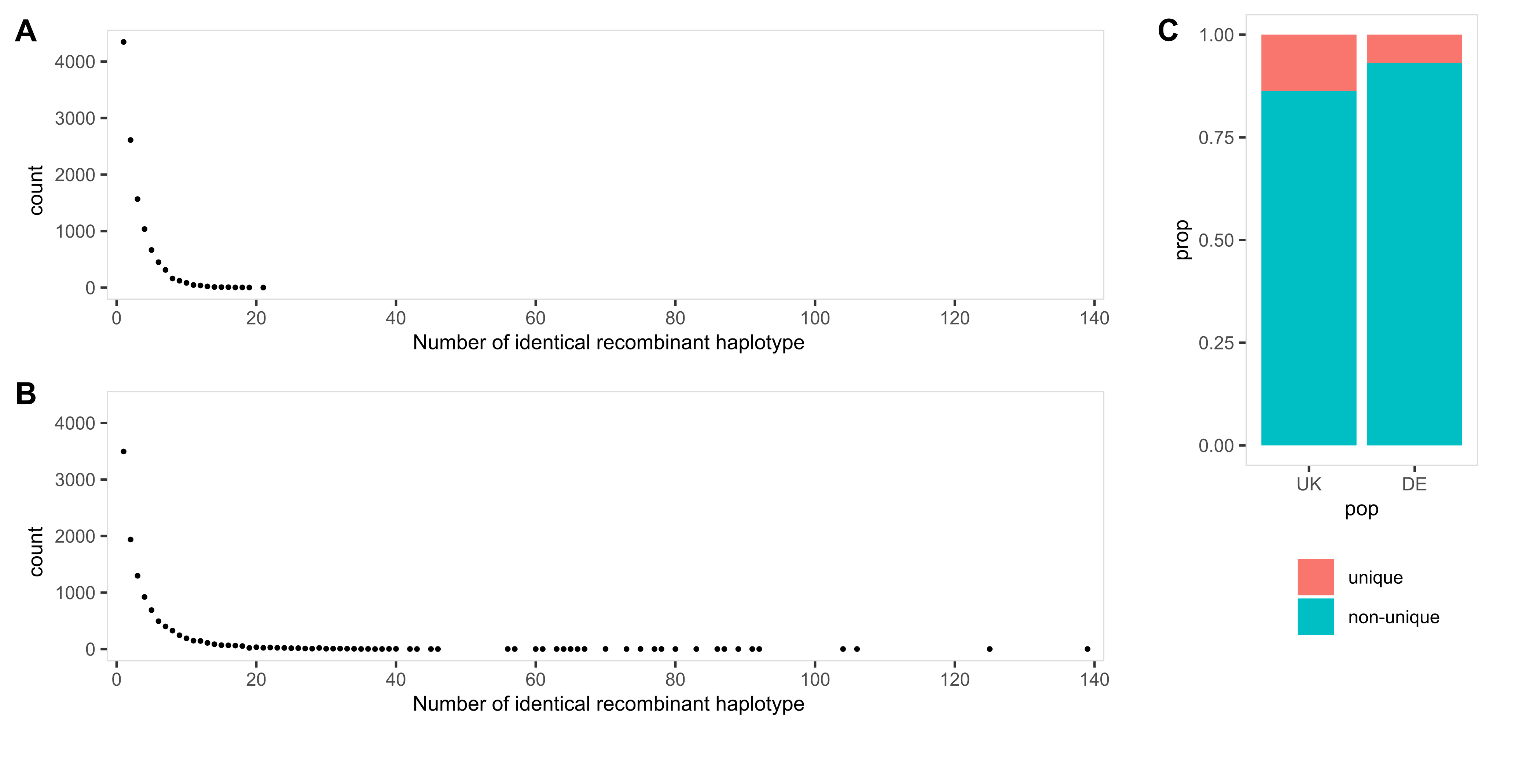


## Figure S3. Distributions of unique and identical recombinant haplotypes in two wheat MAGIC populations.

Recombinant haplotypes are considered identical if they are of the same founder pairs and present in the same 10 cM interval, otherwise unique. [**A**] Counts of the number of identical recombinant haplotypes in wheat-UK8. The left most point is the count of unique recombinant haplotypes. [**B**] Counts of the number of identical recombinant haplotypes in wheat-DE8. [**C**] Proportions of unique and non-unique (identical) recombinant haplotypes in wheat-UK8 and wheat-DE8.


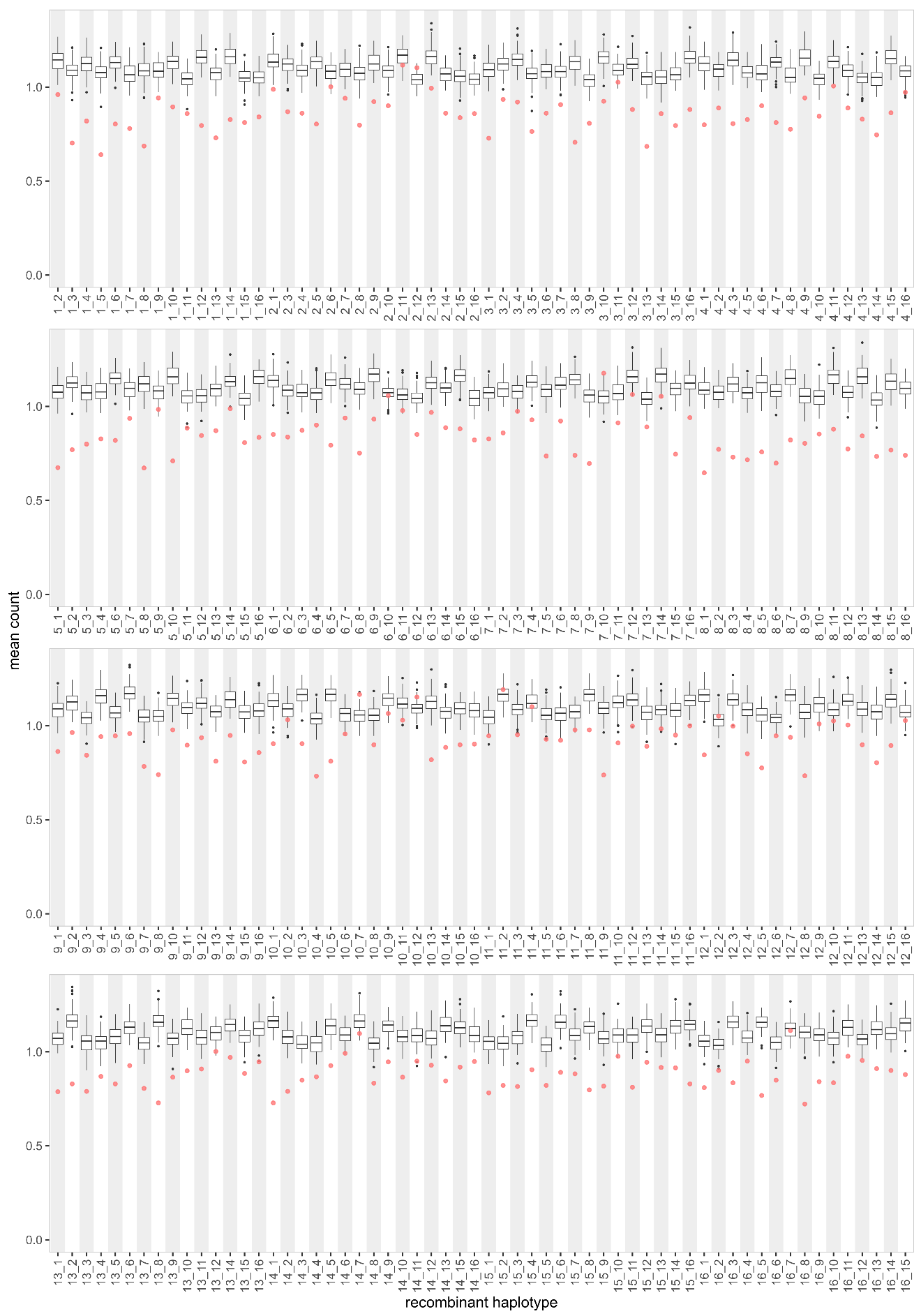


## Figure S4. Distributions of recombinant haplotypes in wheat-UK16.

Plot shows mean count of each recombinant haplotype in a single RIL in wheat-UK16. The boxplot shows mean count from true founder genotypes (100 simulated iterations). The red points show mean count from inferred founder genotypes in actual dataset.

# Literature Cited

Bandillo, N., C. Raghavan, P. A. Muyco, M. A. L. Sevilla, I. T. Lobina *et al*., 2013 Multi-parent advanced generation inter-cross (MAGIC) populations in rice: progress and potential for genetics research and breeding. Rice 6: 11.

Bülow, L., M. Nightingale and L. Frese, 2019 A MAGIC population as an approach to the maintenance and development of breeding usable genetic diversity of winter barley through on-farm management. J. Kulturpflanzen 71: 286-298.

Campanelli, G., S. Sestili, N. Acciarri, F. Montemurro, D. Palma *et al*., 2019 Multi-parental advanced generation inter-cross population, to develop organic tomato genotypes by participatory plant breeding. Agronomy 9: 119.

Dell’Acqua, M., D. M. Gatti, G. Pea, F. Cattonaro, F. Coppens *et al*., 2015 Genetic properties of the MAGIC maize population: a new platform for high definition QTL mapping in *Zea mays*. Genome Biol. 16: 167.

Descalsota, G. I. L., B. P. M. Swamy, H. Zaw, M. A. Inabangan-Asilo, A. Amparado *et al*., 2018 Genome-wide association mapping in a rice MAGIC Plus population detects QTLs and genes useful for biofortification. Front. Plant Sci. 9: 1347.

Gnan, S., A. Priest and P. X. Kover, 2014 The genetic basis of natural variation in seed size and seed number and their trade-off using *Arabidopsis thaliana* MAGIC lines. Genetics 198: 1751-1758.

Han, Z., G. Hu, H. Liu, F. Liang, L. Yang *et al*., 2020 Bin-based genome-wide association analyses improve power and resolution in QTL mapping and identify favorable alleles from multiple parents in a four-way MAGIC rice population. Theor. Appl. Genet. 133: 59-71.

Huang, B. E., A. W. George, K. L. Forrest, A. Kilian, M. J. Hayden *et al*., 2012 A multiparent advanced generation inter-cross population for genetic analysis in wheat. Plant Biotechnol. J. 10: 826-839.

Huang, C., C. Shen, T. Wen, B. Gao, D. Zhu *et al*., 2018 SSR-based association mapping of fiber quality in upland cotton using an eight-way MAGIC population. Mol. Genet. Genom. 293: 793-805.

Huang, X., M.-J. Paulo, M. Boer, S. Effgen, P. Keizer *et al*., 2011 Analysis of natural allelic variation in *Arabidopsis* using a multiparent recombinant inbred line population. Proc. Natl. Acad. Sci. USA 108: 4488-4493.

Huynh, B.-L., J. D. Ehlers, B. E. Huang, M. Muñoz-Amatriaín, S. Lonardi *et al*., 2018 A multi-parent advanced generation inter-cross (MAGIC) population for genetic analysis and improvement of cowpea (*Vigna unguiculata* L. Walp.). Plant J. 93: 1129-1142.

Islam, M. S., G. N. Thyssen, J. N. Jenkins, L. Zeng, C. D. Delhom *et al*., 2016 A MAGIC population-based genome-wide association study reveals functional association of *GhRBB1_A07* gene with superior fiber quality in cotton. BMC Genomics 17: 903.

Jiménez-Galindo, J. C., R. A. Malvar, A. Butrón, R. Santiago, L. F. Samayoa *et al*., 2019 Mapping of resistance to corn borers in a MAGIC population of maize. BMC Plant Biol. 19: 431.

Khazaei, H., F. L. Stoddard, R. W. Purves and A. Vandenberg, 2018 A multi-parent faba bean (*Vicia faba* L.) population for future genomic studies. Plant Genet. Res. 16: 419-423.

Li, D. G., Z. X. Li, J. S. Hu, Z. X. Lin and X. F. Li, 2016 Polymorphism analysis of multi-parent advanced generation inter-cross (MAGIC) populations of upland cotton developed in China. Genet. Mol. Res. 15: gmr15048759.

Li, X.-F., Z.-X. Liu, D.-B. Lu, Y.-Z. Liu, X.-X. Mao *et al*., 2013 Development and evaluation of multi-genotype varieties of rice derived from MAGIC lines. Euphytica 192: 77-86.

Li, Z., G. Ye, M. Yang, Z. Liu, D. Lu *et al*., 2014 Genetic characterization of a multiparent recombinant inbred line of rice population. Res. Crops 15: 28-37.

Mackay, I. J., P. Bansept-Basler, T. Barber, A. R. Bentley, J. Cockram *et al*., 2014 An eight-parent multiparent advanced generation inter-cross population for winter-sown wheat: creation, properties, and validation. G3: Genes, Genomes, Genetics 4: 1603-1610.

Mahan, A. L., S. C. Murray and P. E. Klein, 2018 Four-parent maize (FPM) population: development and phenotypic characterization. Crop Sci. 58: 1106-1117.

Meng, L., L. Guo, K. Ponce, X. Zhao and G. Ye, 2016 Characterization of three *indica* rice multiparent advanced generation intercross (MAGIC) populations for quantitative trait loci identification. Plant Genome 9: 1-14.

Milner, S. G., M. Maccaferri, B. E. Huang, P. Mantovani, A. Massi *et al*., 2015 A multiparental cross population for mapping QTL for agronomic traits in durum wheat (*Triticum turgidum* ssp. *durum*). Plant Biotechnol. J. 14: 735-748.

Novakazi, F., L. Krusell, J. D. Jensen, J. Orabi, A. Jahoor *et al*., 2020 You had me at “MAGIC”!: four barley MAGIC populations reveal novel resistance QTL for powdery mildew. Genes 11: 1512.

Ogawa, D., E. Yamamoto, T. Ohtani, N. Kanno, H. Tsunematsu *et al*., 2018 Haplotype-based allele mining in the Japan-MAGIC rice population. Sci. Rep. 8: 4379.

Ongom, P. O. and G. Ejeta, 2018 Mating design and genetic structure of a multi-parent advanced generation intercross (MAGIC) population of Sorghum (*Sorghum bicolor* (L.) Moench). G3: Genes, Genomes, Genetics 8: 331-341.

Pascual, L., N. Desplat, B. E. Huang, A. Desgroux, L. Bruguier *et al*., 2015 Potential of a tomato MAGIC population to decipher the genetic control of quantitative traits and detect causal variants in the resequencing era. Plant Biotechnol. J. 13: 565-577.

Raghavan, C., R. Mauleon, V. Lacorte, M. Jubay, H. Zaw *et al*., 2017 Approaches in characterizing genetic structure and mapping in a rice multiparental population. G3: Genes, Genomes, Genetics 7: 1721-1730.

Sallam, A. and R. Martsch, 2015 Association mapping for frost tolerance using multi-parent advanced generation inter-cross (MAGIC) population in faba bean (*Vicia faba* L.). Genetica 143: 501-514.

Sannemann, W., A. Lisker, A. Maurer, J. Léon, E. Kazman *et al*., 2018 Adaptive selection of founder segments and epistatic control of plant height in the MAGIC winter wheat population WM-800. BMC Genomics 19: 559.

Sannemann, W., B. E. Huang, B. Mathew and J. Léon, 2015 Multi-parent advanced generation inter-cross in barley: high-resolution quantitative trait locus mapping for flowering time as a proof of concept. Mol. Breed. 35: 86.

Scott, M. F., N. Fradgley, A. R. Bentley, T. Brabbs, F. Corke *et al*., 2021 Limited haplotype diversity underlies polygenic trait architecture across 70 years of wheat breeding. Genome Biol. in press. https://www.biorxiv.org/content/10.1101/2020.09.15.296533v1.

Shah, R., B. E. Huang, A. Whan, M. Newberry, K. Verbyla *et al*., 2019 The complex genetic architecture of recombination and structural variation in wheat uncovered using a large 8-founder MAGIC population. bioRxiv. doi: 10.1101/594317 (Preprint posted March 31, 2019).

Shivakumar, M., G. Kumawat, C. Gireesh, S. V. Ramesh and S. M. Husain, 2018 Soybean MAGIC population: a novel resource for genetics and plant breeding. Curr. Sci. 114: 906-908.

Stadlmeier, M., L. Hartl and V. Mohler, 2018 Usefulness of a multiparent advanced generation intercross population with a greatly reduced mating design for genetic studies in winter wheat. Front. Plant Sci. 9: 1825.

Thépot, S., G. Restoux, I. Goldringer, F. Hospital, D. Gouache *et al*., 2015 Efficient tracking selection in a multiparental population: the case of earliness in wheat. Genetics 199: 609-623.

Wada, T., K. Oku, S. Nagano, S. Isobe, H. Suzuki *et al*., 2017 Development and characterization of a strawberry MAGIC population derived from crosses with six strawberry cultivars. Breed. Sci. 67: 370-381.

Yan, W., H. Zhao, K. Yu, T. Wang, A. N. Khattak *et al*., 2020 Development of a multiparent advanced generation intercross (MAGIC) population for genetic exploitation of complex traits in *Brassica juncea*: glucosinolate content as an example. Plant Breed. 139: 779-789.

Zaw, H., C. Raghavan, A. Pocsedio, B. P. M. Swamy, M. L. Jubay *et al*., 2019 Exploring genetic architecture of grain yield and quality traits in a 16-way *indica* by *japonica* rice MAGIC global population. Sci. Rep. 9: 19605.

Zhao, F.-Y., H. Zhao, X.-L. Wang and X.-F. Li, 2017 Construction and application potential of MAGIC population on genetic breeding of rapeseed (*Brassica napus* L.). Chinese J. Oil Crop Sci. 39: 149-151.
